# Antibacterial Property of Cellulose Acetate Composite Materials Reinforced with Aluminum Nitride

**DOI:** 10.3390/antibiotics10111292

**Published:** 2021-10-22

**Authors:** Thefye P. M. Sunthar, Francesco Boschetto, Hoan Ngoc Doan, Taigi Honma, Kenji Kinashi, Tetsuya Adachi, Elia Marin, Wenliang Zhu, Giuseppe Pezzotti

**Affiliations:** 1 Ceramic Physics Laboratory, Kyoto Institute of Technology, Sakyo-ku, Matsugasaki, Kyoto 606-8585, Japan; boschetto-cesc@kit.ac.jp (F.B.); m0672026@edu.kit.ac.jp (T.H.); elia-marin@kit.ac.jp (E.M.); wlzhu@kit.ac.jp (W.Z.); pezzotti@kit.ac.jp (G.P.); 2 Department of Immunology, Graduate School of Medical Science, Kyoto Prefectural University of Medicine Kamigyo-ku, 465 Kajii-cho, Kawaramachi dori, Kyoto 602-0841, Japan; 3 Department of Dental Medicine, Graduate School of Medical Science, Kyoto Prefectural University of Medicine, Kamigyo-ku, Kyoto 602-8566, Japan; t-adachi@koto.kpu-m.ac.jp; 4 Faculty of Material Science and Engineering, Kyoto Institute of Technology, Sakyo-ku, Matsugasaki, Kyoto 606-8585, Japan; ngochoandoan@gmail.com (H.N.D.); kinashi@kit.ac.jp (K.K.); 5 The Center for Advanced Medical Engineering and Informatics, Osaka University, Yamadaoka, Suita, Osaka 565-0871, Japan; 6 Department of Orthopedic Surgery, Tokyo Medical University, 6-7-1 Nishi-Shinjuku, Shinjuku-ku, Tokyo 565-0871, Japan

**Keywords:** aluminum nitride, composite, antibacterial, mechanical, thermal, cellulose acetate

## Abstract

Cellulose acetate (CA) is a synthetic compound that is derived from the acetylation of cellulose. CA is well known as it has been used for many commercial products such as textiles, plastic films, and cigarette filters. In this research, antibacterial CA composites were produced by addition of aluminum nitride (AlN) at different weight percentage, from 0 wt. % to 20 wt. %. The surface characterization was performed using laser microscope, Raman and FTIR spectroscopy. The mechanical and thermal properties of the composite were analyzed. Although the mechanical strength tended to decrease as the concentration of AlN increased and needed to be optimized, the melting temperature (T_m_) and glass transition temperature (T_g_) showed a shift toward higher values as the AlN concentration increased leading to an improvement in thermal properties. AlN additions in weight percentages >10 wt. % led to appreciable antibacterial properties against *S. epidermidis* and *E. coli* bacteria. Antibacterial CA/AlN composites with higher thermal stability have potential applications as alternative materials for plastic packaging in the food industry.

## 1. Introduction

Food provides nutritional support for any organism. However, most people around the world are not aware of the dangers related to the lack of proper food hygiene. According to a survey from World Health Organization (WHO), 600 million cases of foodborne diseases and 420,000 deaths were recorded worldwide in the year 2015. The common bacteria that responsible for the foodborne illness are *Norovirus*, *Listeria*, *Campylobacter jejuni*, *Salmonella*, *Staphylococcus aureus*, *Escherichia coli* [1,2].

Food contamination may happen during harvesting, processing, packaging and distribution [2]. However, packaging plays an important role to protect food from being affected by various kinds of contaminants and preserve the products from biological, chemical and physical changes while storage or during preparation. In many so-called “advanced countries”, the food quality is a very important factor—they used to reject it even if there was a small change in the smell or appearance [1].

In literature, many different techniques were applied to improve the antimicrobial properties of packaging systems [2]. Some of the methods are based on the reinforcement of volatile and nonvolatile antimicrobial compounds directly into the polymers like the application of sachet or pads that contain volatile antimicrobial compounds, applying antimicrobial coating compounds on the surface of the polymers, ions or covalent linkages of immobilization of antimicrobial agent into the polymers [3,4,5]. In other cases, chitosan was directly used without any modification as an antimicrobial coating film [3,4,5,6]. After consideration of the previous research, this research is aimed to implement the new and easy idea to produce the antibacterial composite material using easily degradable cellulose acetate material as the main substrate and reinforced with AlN which could replace the usage of plastic material.

Cellulose acetate (CA) is a synthetic compound which is derived from the acetylation of cellulose. Cellulose is a natural polymer obtained from plant fibers, with the chemical formula C_6_H_7_O_2_ (OH)_3_ [7]. However, pure cellulose has a complex structure that cannot be easily modulated by using heat or solvents. The acetylation process causes the hydrogen in the hydroxyl groups is replaced by acetyl groups (CH_3_CO) which turns it into cellulose acetate. CA is easier to dissolved in certain solvents like acetone or can be melted under heat and molded into solid objects, spun into fibers or cast as a film [8].

Aluminum nitride (AlN) is well known for its excellent thermal conductivity, high coefficient of thermal expansion, high electrical resistivity and high dielectric strength [9]. Besides the wide use of AlN in semiconductors, it showed antimicrobial properties such as those of Si_3_N_4_ [10]. This property is due to the reactivity of the AlN with water which produces ammonia (NH_3_) and ammonium ions (NH_4_^+^) that eventually kill the bacteria [10].

This research focuses on producing alternative food packaging materials, plastic tablecloths or toys by reinforcing CA with AlN. The CA/AlN composites can be produced with a lower cost and non-complicated processes. In addition, it also can show both mechanical and thermal stability with enhanced antibacterial property.

Possible future developments include degradable CA/AlN composites which might contribute to the reduction of environmentally harmful plastic waste.

## 2. Materials and Methods

### 2.1. Samples Preparation

**Figure 1:**
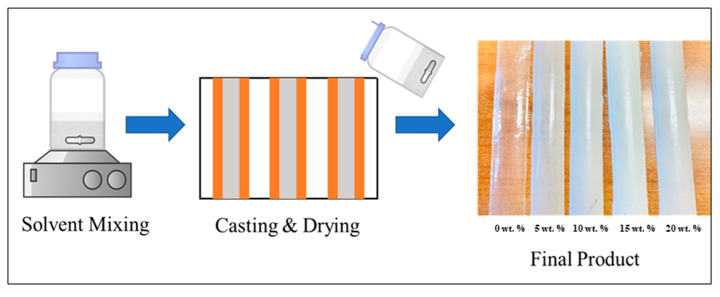
Experimental procedure of CA/AlN composites.

The samples were prepared as showed in Figure 1. Cellulose acetate powder was crushed using pestle and mortar to obtain fined powder. Then the powder was mixed with acetone until completely dissolved and 0.05 mL of pure triacetin were mixed to the solution, as a plasticizer. Once the solution cleared, agglomerated AlN powder were poured into the glass vial and mixed by stirring until a homogenous mixture is obtained. The liquid is then poured inside a flat mold obtained by using polyimide tape on the Teflon plate. Then the film was casted using glass plate to fill the entire row to form a layer. The casting process were repeated 5 times to obtain a desire thickness. The film was left overnight to dry at room temperature. The film was removed from the plate after the acetone evaporated and forming a layer of the CA/AlN composites. It was then heated at 60 °C in a vacuum for overnight to remove the remaining solvent inside the film. Five different samples were produced with 0 wt. %, 5 wt. %, 10 wt. %, 15 wt. % and 20 wt. % of AlN, respectively, (i.e., CA content = 100 wt. % − AlN wt. %).

### 2.2. Sample Characterization

#### 2.2.1. Laser Microscopy

The surface morphology of the sample was analyzed with the aid of a confocal scanning laser microscope (Laser Microscope 3D and Profile measurements, Keyence, VK × 200 Series, Osaka, Japan) equipped with a numerical aperture between 0.30 and 0.95. It has the x-y stage and autofocus function for z range. The micrographs were collected ranging from 10× to 150× to evaluate macroscopic and microscopic roughness of the samples. 25 images randomly selected from the surface of the map and the micrographs were then analyzed using Keyence Color 3D Laser Microscope VK-X100/X200 series VK Analyzer software (Keyence, Osaka, Japan).

#### 2.2.2. Fourier Transformed Infrared Spectroscopy

Fourier Transformed Infra-Red spectroscopy (FTIR) spectra were collected at room temperature using an FTIR spectrometer (ATR-FTIR, FTIR-4700 with ATR PRO ONE equipped with a diamond prism, Jasco Co., Tokyo, Japan) with a Michelson 28-degree interferometer with corner-cube mirrors with a range between 250,000 and 5 cm^−1^. The aperture size was 200 × 200 µm^2^ the acquisition time was fixed to 30 s. The instrument was operated using (Spectra Manager, JASCO, Tokyo, Japan) software. A total of three samples of each type were scanned from 400 and 4000 cm^−1^ at 5 different locations. The spectra were analyzed with (OriginLab Co., Northampton, MA, USA, and LabSpec, Horiba/Jobin-Yvon, Kyoto, Japan) software.

#### 2.2.3. Raman Spectroscopy

Raman spectra of the samples were collected with the aid of triple monochromator (T-64000, Jobin-Ivon/Horiba Group, Kyoto, Japan) equipped with a charge coupled device (CCD) detector. The excitation source used is 532 nm Nd:YVO4 diode pumped solid-state laser (SOC JUNO, Showa Optronics Co. Ltd., Tokyo, Japan). In total, 25 randomly picked locations were investigated with spectrograph center wavelength 2500 cm^−1^, grating 300 gr/mm, exposure time 4 s and average of 3. The resulting spectra were averaged. Raman spectral acquisition and pre-processing of raw data such as baseline subtraction, smoothing, normalization and fitting were acquired utilizing commercially available software (LabSpec, Horiba/Jobin-Yvon, Kyoto, Japan and Origin 8.5, OriginLab Co., Northampton, MA, USA).

### 2.3. Mechanical and Thermal Properties

#### 2.3.1. Mechanical Properties

Tensile mechanical testing was conducted using a MCT 2150 Desktop Tensile-Compression Tester (AND Discover Precision, Tokyo, Japan) using a 500 N load cell at a strain rate of 50 mm min^−1^. For testing, CA/AlN composite samples were cut using a standardized dumbbell shaped tensile sample cutter with an overall length of 35 mm, gauge length of 10 mm, distance between shoulders 12 mm, grip section 4.5 mm, width of grip section 6 mm, reduced section 12 mm. In total, 5 samples were tested with each concentration (0, 5, 10, 15, and 20 wt. %). The result was analyzed using Excel and OriginLab (Co., Northampton, MA, USA).

#### 2.3.2. Thermal Properties

The thermal properties of the CA composite were investigated using a differential scanning calorimeter (DSC) (TA Q200, TA Instruments Japan Inc., Tokyo, Japan) with a heating/cool/heat cycle program. The sample was heated at 10 °C min^−1^ from −30 to 300 °C and cooled at 5 °C min^−1^ under a nitrogen atmosphere with a gas flow rate of 50 μL·h^−1^. Each sample was measured three times.

### 2.4. In Vitro Testing 

The antibacterial analysis was conducted at the Kyoto Prefectural University of Medicine. Gram-positive bacteria, *Staphylococcus epidermidis* (14990^TM^ ATCC^®^ purchased from American Type Culture Collection (ATCC)) and Gram-negative bacteria, *Escherichia coli* (*E. coli*, ATCC^®^ 25922™)*,* were cultured using a brain heart infusion (BHI) liquid medium. The initial 1.8 × 10^10^ CFU/mL was subsequently diluted to 1.8 × 10^8^ CFU/mL using a phosphate-buffered saline (PBS, NACALAI TESQUE.INC, Kyoto, Japan) solution to mimic ion blood concentrations. The samples with dimensions of 1 cm × 1 cm were sterilized prior to the experiment using a UV sterilizer for 24 h. Then the samples were incubated at 37 °C for 12 and 24 h.

#### 2.4.1. Microbial Viability Assay (WST)

WST is well known technique to measure the bacterial metabolism by calorimetric detection. In this experiment, the WST-8 kit (Microbial Viability Assay Kit-WST, Dojindo, Kumamoto, Japan) was used as a calorimetric indicator which releases a water-soluble formazan dye upon reduction in the presence of electron mediator. The amount of the formazan dye generated is linearly related to the number of living microorganisms. The solution is subjected to microplate readers (EMax, Molecular Devices, Sunnyvale, CA, USA) upon collecting the OD value related to living cells. Three samples were used to calculate the average values.

#### 2.4.2. Crystal Violet Assay, Laser Microscopy Scanning, Viability Staining and ImageJ Analysis

A 0.5% crystal violet solution was used to determine the biofilm formation on the sample’s surface. The PBS washed samples were placed into a 12-well plate filled with 500 μL crystal violet solution and incubated at room temperature for 20 min on shaker. The samples were then transferred to a new 6-well plate and washed four times gently with 5 mL PBS to excrete excess crystal violet. The plates were gently shaken to completely remove the residual dye. Then, the samples were measured using a laser microscope to measure the bacterial attachment and biofilm formation on the sample surface. The laser micrographs are then analyzed with ImageJ 1.50, to measure the area filled by bacteria. The analysis of biofilm density was measured by conversion of confocal images into red, green and blue colors. The background subtraction was applied to remove background noise. The images were then assembled into color channels and the integrated density of pixels was calculated. Correspondingly, the same samples were used to measure the OD of the samples with crystal violet stained which represent the amount of biofilm. The samples were transferred into a new 12-well plate filled with 95% ethanol and the plate was incubated at room temperature on a shaker at 270 rpm for 15 min. Then, 100 μL of the ethanol solution containing the crystal violet stained by the biofilms was transferred into the 96-well plate. The optical density at 595 nm was determined for total biomass quantification. 

### 2.5. Statistical Analysis

The experimental data were statistically analyzed with Student’s t-test (t-test) where *p* < 0.05 was considered statistically significant and marked with an asterisk (*) and not statistically significant data marked with (*ns*).

## 3. Results

### 3.1. Surface Characterization

#### 3.1.1. Laser Microscopy

**Figure 2:**
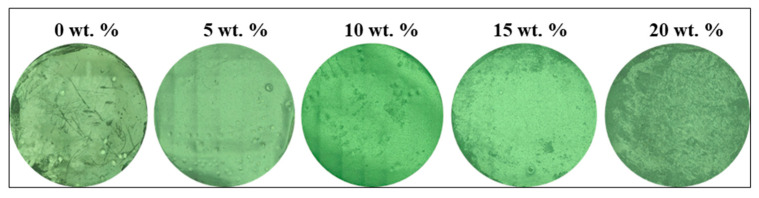
Laser microscope images of CA/AlN composites.

Figure 2 shows the laser micrographs images of five samples with different concentration of AlN ranging from 0 to 20 wt. %. The dispersion of AlN on the surface could be clearly observed as a function of concentration. 

**Figure 3:**
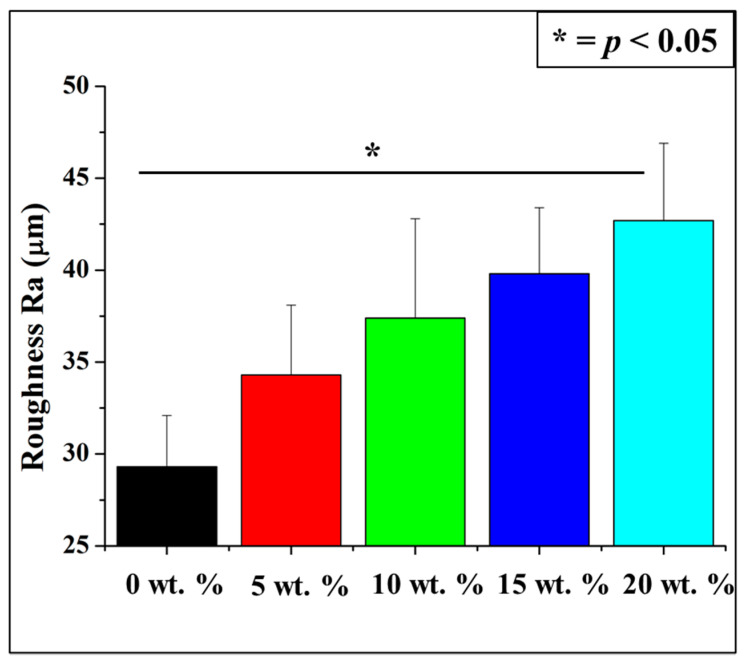
Surface microscopic roughness of CA/AlN composites. * = *p* < 0.05.

The white particles observed are the AlN. The surface roughness R_a_ increases as the amount of AlN increases. Figure 3 shows the quantification of surface roughness where a clear increasing trend with AlN concentration was observed. As shown, 20 wt. % shows the highest surface roughness and the whitish particle observed in image is AlN which increases the surface roughness of the material.

#### 3.1.2. FTIR Spectroscopy

**Figure 4:**
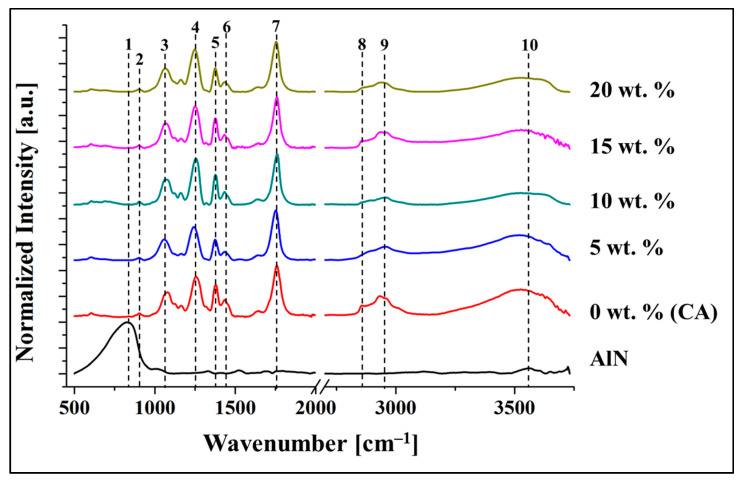
FTIR spectra of the CA/AlN composites.

Figure 4 shows the FTIR spectra of the pure aluminum nitride, and the CA/AlN composites ranging from 0 wt. % to 20 wt. %. The composite material has a wide range spectrum from 500 to 3750 cm^−1^. The FTIR spectra do not shows the presence of AlN as it can be clearly observed that none of the CA/AlN composites has the strong peak of AlN at 672 cm^−1^ (peak 1) which is assigned to E1(TO) [11,12,13]. Peak 2 to 10 represents cellulose acetate functional groups. The band at 1750 cm^−1^ (peak 7) was attributed to C=O from cellulose acetate and the band at 1428 cm^−1^ (peak 6) was assigned to CH_2_ vibrations. The sharp absorption peaks at 1041 cm^−1^ (peak 3) and 1250 cm^−1^ (peak 4) were due to presence of C-O stretching [14,15]. The band at 912 cm^−1^ (peak 2) can be attributed to C-O stretching and CH_2_ rocking vibrations. In addition, 1366 cm^−1^ (peak 5) was assigned to CH_3_. The broad peak at and 2942 cm^−1^ (peak 9) and the peak at 3487 cm^−1^ (peak 10) attributed to C-H aromatic vibrations and O-H stretching of cellulose acetate [16,17], respectively. FTIR do not show any functional groups of AlN which are present in the CA/AlN composites. This might be because the strong signal of CA blocks the weak signals of AlN in the composite.

#### 3.1.3. Raman Spectroscopy

**Figure 5:**
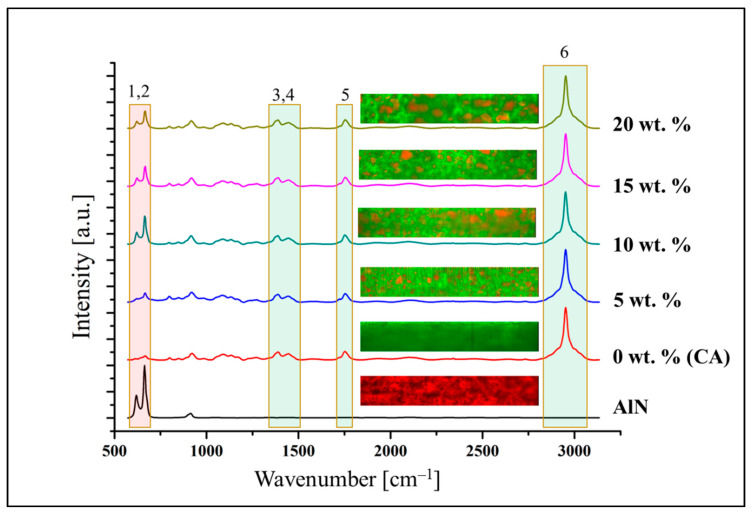
Raman average spectra of the CA/AlN composites.

The functional groups of CA could by clearly observed by FTIR, while Raman spectroscopy was used to further investigate both the CA/AlN composites and AlN. The various spectra acquired were illustrated in Figure 5. The characteristics of Raman signal for cellulose was clearly observed at 2934 cm^−1^ (peak 6) which is assigned to C-H stretching and asymmetric stretching vibrations of the C-O-C glycosidic linkage. In addition, Raman signals at 1380 (peak 3), 1435 (peak 4) and 1754 cm^−1^ (peak 5) are attributed to C=O vibrations of the carbonyl group and asymmetric and symmetric vibrations of C-H bond which exist in the acetyl group from CA [17,18,19]. Besides, the presence of AlN could be clearly observed at 612 (peak 1) and 656 cm^−1^ (peak 2) which are associated with A_1_ (TO) and E_2_ (high) [20,21,22]. As AlN concentration increases the peak intensity also increases. The red spots are a marker for the presence of the AlN in the CA matrix, which is green. The intensity of the red spot increases in conjunction with the peak intensity, which can be explained with the increased presence of AlN. The AlN signals, which cannot be detected by FTIR, were clearly seen in the Raman spectra which proves the presence of AlN particles in the composite. 

### 3.2. Mechanical Property

**Figure 6:**
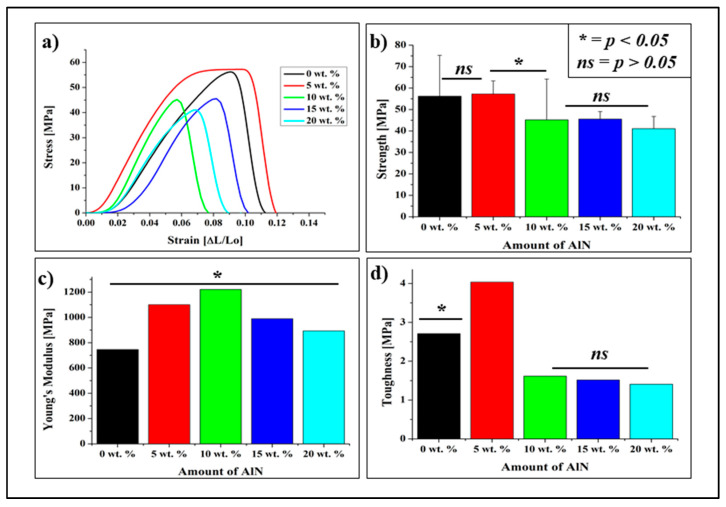
(**a**). Tensile stress-strain curves of the CA/AlN composites, (**b**) Max strength, (**c**) Young’s modulus and (**d**) Toughness. * = *p* < 0.05, *ns* = *p* > 0.05.

Figure 6a,b illustrates the stress versus strain curves for the various CA/AlN composites. It provides the information on how the composite materials deforms with increasing force. The composites containing 5 wt. % of AlN has similar strength to the 0 wt. %. Mechanical strength was then reduced in the samples containing 10 wt. % to 20 wt. %. This proves that 5 wt. % reinforcement concentration has the highest strength among the composite samples.

Figure 6c shows the young modulus of the CA/AlN composites which were calculated using gradient of the stress versus strain curves. The Young’s modulus of the samples containing 5 wt. % and 10 wt. % of AlN increases up to 1100 and 1221 MPa, respectively. However, when the further AlN were added, the Young’s modulus of the 15 wt. % and 20 wt. % samples decrease to 989 and 892 MPa, respectively Therefore, it can be deduced that the stiffness of the samples increases with low AlN concentration (5 wt. % and 10 wt. %) and then reduce when the AlN reached to 15 and 20 wt. %.

In addition, the changes in toughness of the material were calculated by area un-der the graph as shown in Figure 6d. The toughness of the 0 wt. % sample was 2.7 MPa. When 5 wt. % of AlN was added to the composite, the toughness significantly increased, up to 4.0 MPa. which shows it is toughest sample. However, the toughness started to reduce when higher AlN concentrations were added to the composites. The calculations analysis was made as stated in the literature [18,23,24].

HDPE, PVC, and PVDC are the commercially available material which is used to produce cling film for food packaging, tablecloths and other plastic materials. Theoretically, the commercial HDPE, PVC and PVDC clings film has the tensile strength of ≥10, ≥15 and ≥60 MPa respectively. Therefore, the CA/AlN composites can be used as the food wrap or tablecloths since it has the tensile strength in the range of 57 to 41 MPa which meets the range of tensile strength of commercial product. As a summary, though the tensile strength reduces at 20 wt. % but it has the enough mechanical strength to be an alternative material when compared with the theoretical values of the commercial products. However, 20 wt. % showed high thermal and antibacterial property which enhance the composite material’s efficiency.

### 3.3. Thermal Properties

**Figure 7:**
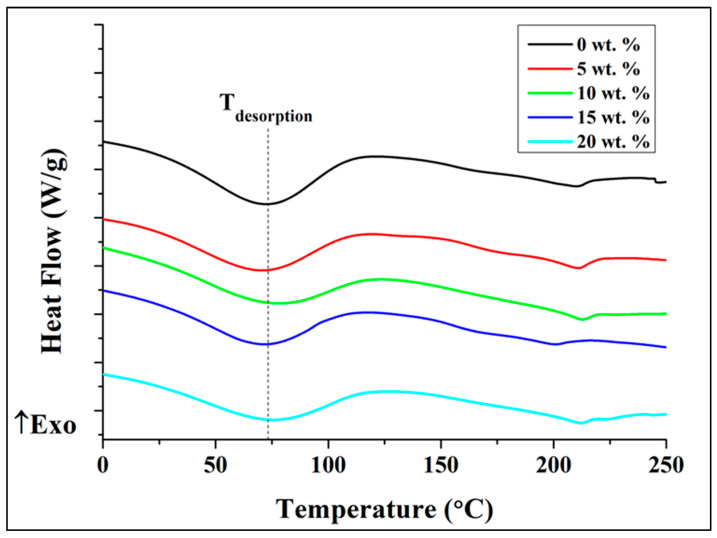
DSC first heating curves for the CA/AlN composites.

The thermal property of the CA/AlN composites was analyzed by differential scanning calorimetry, DSC. The desorption temperature (T_d_), glass transition temperature (T_g_), melting temperature (T_m_), and degree of crystallinity χ*_c_* were measured. Figure 7 shows the first heating curve of DSC of cellulose acetate and CA/AlN composites. The first endothermic peak was clearly seen in all the samples. This peak appears due to the desorption of water. This phenomenon occurs due to the existence of residual moisture or low boiling point of solvents. T_d_ varies from control (0 wt. %) to 20 wt. % in the range from 71.82 °C to 79.97 °C. The differences of T_d_ values are due to their different ability of holding water of the polymeric matrices [14]. 

**Figure 8:**
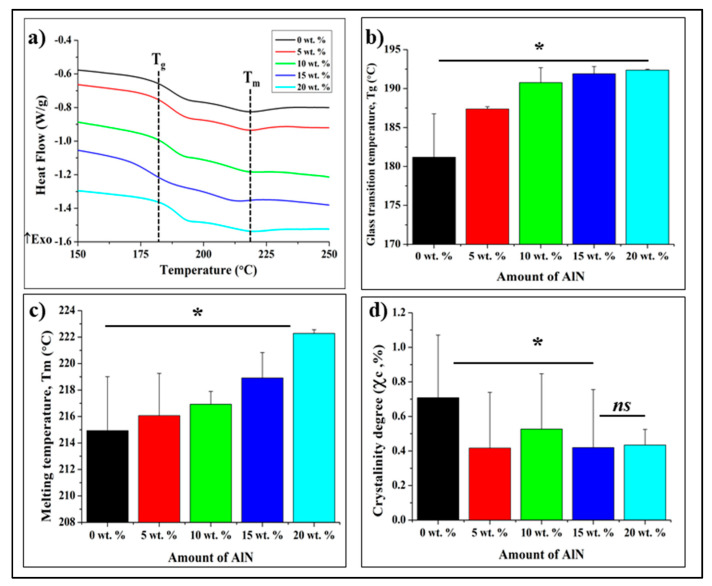
DSC curves for the CA/AlN composites (**a**), 2nd heating curve (**b**), Glass transition temperature, Tg, (**c**), Melting temperature and (**d**), crystallinity degree. * = *p* < 0.05, *ns* = *p* > 0.05.

Figure 8a shows the second heating curve where the T_g_ and T_m_ could be identified. The T_g_ value were calculated by the maximum Tan delta peak [25,26,27,28,29]. The T_g_ values increases as the concentration of AlN increases as illustrated in Figure 8b. The T_g_ value of the CA improved with increasing the amount of AlN reinforcement.

The T_m_ temperature also shows similar trend where it increases as the amount of AlN increases. However, the increment seems to be small (about 7.35 °C from 0 wt. % to 20 wt. %). From Figure 8c, an endothermic peak appears in the first cycle close to the melting peak, but according to literature [26,29,30], the first heating curve is influenced by the thermal and mechanical history, and the second heating curve is conventionally used for the determination of material properties, such as meting point or glass transition temperature, under a given dynamic condition. For this reason, the melting point and glass transition temperature were investigated using the second heating curve [25,26,27,28,29,30,31,32].

Another parameter extrapolated from the DSC curves is the degree of crystallinity χ*_c_* which is a fundamental property for properties of plastics. A higher degree of crystallization makes the material stiffer and stronger but also increases brittleness [26,27,28,29,30,31]. The crystallinity degree of CA/AlN composites was calculated as the ratio between the melting enthalpy of ($\Delta H_{m}$) and the respective value for the totally crystalline material ($\Delta H_{m}^{0}$) multiply with weight fraction of CA using the following formula:

(1) $$\chi_{c}=\frac{\Delta H_{m}}{\omega \Delta H_{m}^{0}}\times 100$$

where $\Delta H_{m}^{0}$ = 58.8 J g^−1^ as stated by Cerquiera et al. [27].

Based on Figure 8d the degree of crystallinity reduces upon addition of AlN. However, the concentration of AlN does not affect the crystallinity degree much as the value was almost similar from 5 wt. % to 20 wt. %

### 3.4. In Vitro Testing

#### Microbial Viability Assay (WST)

**Figure 9:**
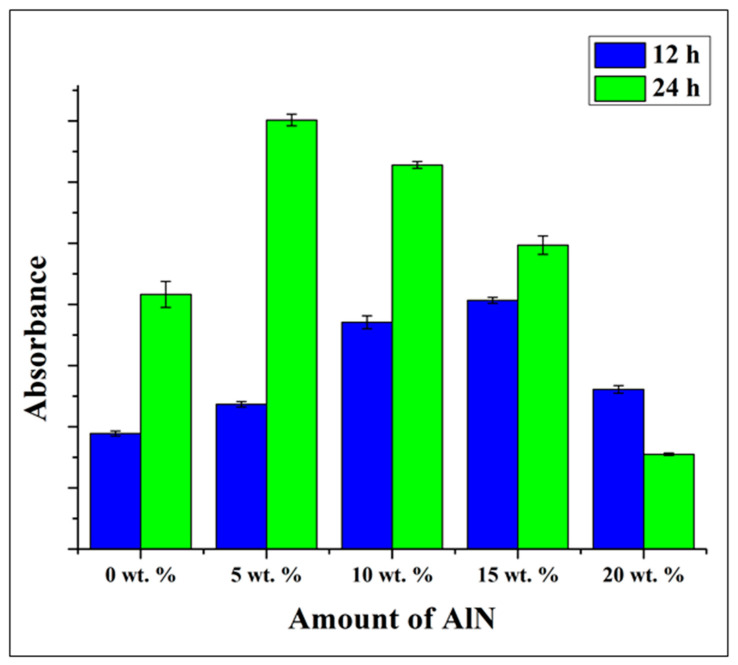
WST adsorption after 12 and 24 h of testing with *Staphylococcus epidermidis* on the CA/AlN composites, as a function of the fraction of AlN.

Figure 9 demonstrates the antibacterial test conducted against the CA/AlN composites material with *S. epidermidis* using WST method. It is clearly observed that absorbance level increases until 15 wt. % and start to reduce at 20 wt. % for 12 h. Besides, the same samples were tested with the laser microscope, where the biofilm production was clearly seen on the surface as shown in Figure S1. Almost similar results were obtained for 12 h, the biofilm formation reduces slightly from the control sample and the antibacterial effect was not clearly observed. Whereas, for 24 h, the absorption shows the similar trend as 12 h, but at 20 wt. % shows a very lower absorption than control. The biofilm formation shows a very clear trend at 24 h where, at control it was observed the full area covered by patches of biofilm and it start to reduce as the concentration of AlN increases and at 20 wt. % and very low amount of biofilm was detected. 

To reconfirm the antibacterial property, the samples were analyzed using Crystal violet (CV) staining method. The stained samples were analyzed with WST and Viability Staining, and ImageJ Analysis and the results were shown in Figure 9 The CV-stained samples show high absorbance at 12 h and start to reduce slightly at higher concentration especially at 15 and 20 wt. %, at 24 h the absorbance does not increase and was maintained as 0 wt. % sample but at 20 wt. % it decreases drastically. When compare with viability staining method, it shows the similar trend as the previous WST method where the biomass increases up to 15 wt. % and decreases at 20 wt. % for 12 h, whereas, at 24 h the biomass shows slight increment up to 15 wt. % and drastic drop at 20 wt. %.

**Figure 10:**
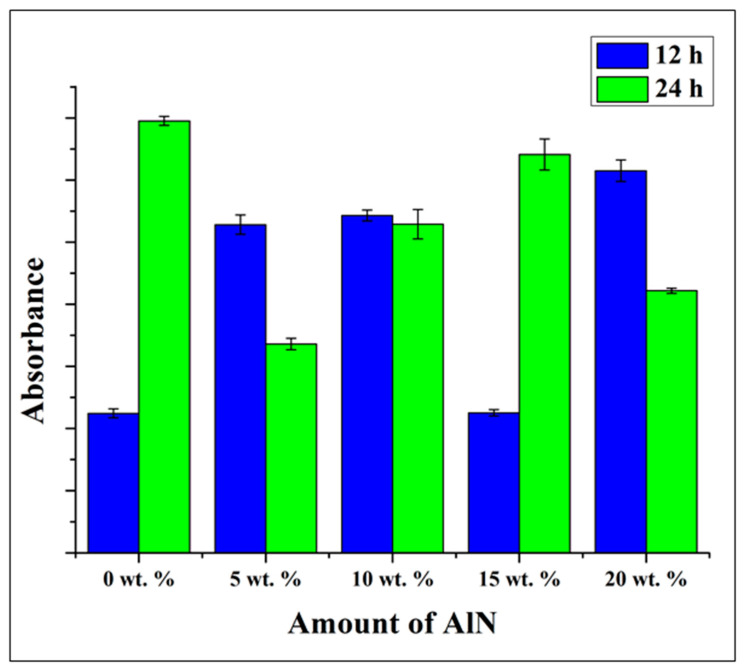
WST adsorption after 12 and 24 h of testing with *Escherichia coli* on the CA/AlN composites, as a function of the fraction of AlN.

For the *E. coli* bacteria, the WST measurement was not precise as the absorbance increases at 12 and 24 h even at 20 wt. % as shown in Figure 10. However, at 24 h, clear trend of decrement was observed especially at 20 wt. %. Comparing with the result of biofilm formation, the same trend was observed where the biofilm increases as the concentration increases. However, at 24 h the reduction of biofilm was clearly observed at 20 wt. % which in agreement with WST result. Although, laser micrographs images in Figure S2 (Supplementary Materials) shows the reduction of the biofilm formation as the concentration of AlN increases at 24 h.

**Figure 11:**
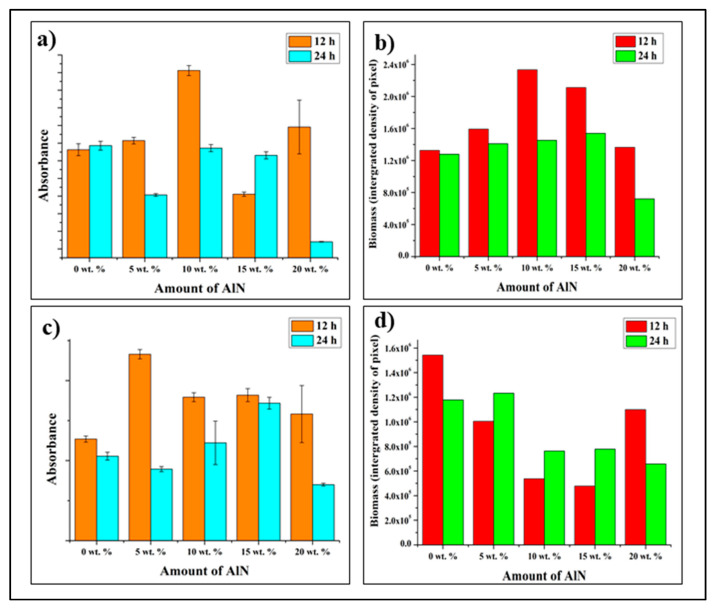
(**a**) *Staphylococcus epidermidis* biofilm formation after 12 and 24 h evaluated by quantitative measurement of crystal violet staining as an indicator of biomass accumulation on CA/AlN composites, (**b**) Quantitative comparison of accumulated biomass in (**a**), (**c**) *Escherichia coli* biofilm formation after 12 and 24 h evaluated by quantitative measurement of crystal violet staining as an indicator of biomass accumulation on CA/AlN composites, (**d**) Quantitative comparison of accumulated biomass in (**c**).

In CV-stained experiment, the absorbance of *E. coli* increases as the concentration increases even at 20 wt. % as illustrated in Figure 11c,d. However, the absorbance shows a decrement at 20 wt. % compared with other concentrations. In the viability test, the biomass of *E. coli* reduces as the concentration increases but shows sudden spike at 20 wt. % for 12 h. However, at 24 h, a clear trend was observed where the biomass starts to reduce after 5 wt. % and lowest at 20 wt. %.

## 4. Discussion

The results of the laser microscope, FTIR and Raman spectroscopy clearly show the surface morphology and chemical composition of the CA/AlN composites. The surface roughness increases as a function of AlN concentration. Raman spectra clearly showed the presence of AlN particles which were hard to observe by FTIR. There was no observable alteration in the chemical bonds of both CA and AlN during the casting process which were clearly seen from FTIR and Raman spectroscopy.

The mechanical properties of the composites are affected by AlN concentration: the ultimate strength, the elongation and the toughness increase with a low concentration of AlN of 5 wt. %, then decreases with AlN concentration of 10 wt. % and above. The Young modulus of the composite also increases with AlN contents of 5 and 10 wt. %, then decreases when the AlN concentrations reach 15 and 20 wt. %. The mechanical behaviors of the CA/AlN composites could be explained by the phenomenon called “mechanical per-collation” [33]. Generally, in the polymer/filler composite system, the mechanical properties of the composite will increase until the filler concentration reaches a critical value, and then decreases with further filler content. The high concentration of the AlN particles could lead to the formation of agglomerates in the polymer matrix, affect the homogeneity of the CA/AlN composites, and causing lower mechanical properties.

The thermal properties were affected by the presence of reinforcing powders: the T_m_ and T_g_ values shows an increment. These increments could be attributed to the presence of AlN particles in the composite system. Due to the agglomeration of the filler particles, the mobility of the polymer chains is reduced. In order to mobilize the polymer chains, more energy is required, leading to the increase in the T_m_ and T_g_ values [34]. Whereas the degree of crystallinity of the composite material reduces when there is presence of AlN. However, the concentration does not affect the degree of crystallinity. The value was almost similar from 5 wt. % to 20 wt. %. Most importantly, this shows AlN can be a good reinforcing material.

The antibacterial properties were clearly shown in the Results chapter; however, it was difficult to observe the effect played by the increasing concentration of AlN which due to the releasing of active antibacterial component from the substrate. At higher concentrations, the antibacterial effect for both Gram-positive (*S. epidermidis)* and Gram-negative (*E. coli*) at 24 h was clearly observed. Secondly, the surface roughness at 20 wt. % might be the reason of the antibacterial effect which is expected to the liberation of ammonia (NH_3_) from the surface of composite material into the aqueous solution upon exposing to water. The high surface roughness promotes more surface area which causes the bacteria to become exposed to the AlN and die. In total, 1 mole of AlN reacts with 3 mole of water and produces 1 mole of aluminum hydroxide and 1 mole of (NH_3_). This reaction was considered as the overall hydrolysis reaction as given below [11,31,32,35,36,37,38]:

AlN + 3H_2_O → Al (OH)_3_ + NH_3_ (2)

However, according to Bowen et al. [39] the hydrolysis of AlN in room temperature, the reaction can be classified into three processes as stated in following equations:

AlN + 2H_2_O → AlOOH_(amorphous)_ + NH_3_ (3)

NH_3_ + H_2_O ⇌ NH^+^_4_ + OH^−^ (4)

AlOOH_(amorphous)_ + H_2_O → Al (OH)_3_ (5)

The release of ammonia reacts with the water to produce ammonium ions and hydroxide ions. The ammonium ions and ammonia release are responsible for the antibacterial property for the composite. There are few studies that have shown the antimicrobial property of ammonium salts. On the other side, the volatile ammonia (NH_3_) gas release is expected to directly attack the structure of DNA of microorganisms [40,41,42,43]. Kleiner focused on the review of transportation of ammonia in bacteria and fungi which explains why bio membranes are highly permeable to free ammonia [39]. In another comprehensive study, the author claimed the ammonium ion, (NH_4_^+^) can only diffuse into the cytoplasmic space through ion channels and the tiny (NH_3_) molecules can freely penetrate through the membrane [36,40,41,42,43]. Therefore, based on the previous studies and the results obtained, it can be speculated that the mechanism of antibacterial action is the elution of ammonia (NH_3_) and ammonium ion (NH_4_^+^) during hydrolysis of AlN, as shown in Equations (1)–(3), diffuses into the bacterial cell and damages the DNA as well as causing cell lysis [35,36,37,38,39,40,41,42,43].

The amount of ammonium ion (NH_4_^+^) and ammonia (NH_3_) released will determine the level of toxicity, thus a small preliminary experiment was conducted to measure the amount of NH_4_^+^ and NH_3_ using a Quick Ammonia Meter AT-2000 instrument and the results were shown in Figure S3. The experiment was conducted at different time intervals of 2, 6, 12 and 24 h using the 20 wt. % of CA/AlN composite. The results demonstrate the release of NH_4_^+^ and NH_3_ are very limited, and even at 24 h the maximum level of NH_4_^+^ and NH_3_ are 0.44 mg/L and 0.28 mg/L respectively. Figure S4 shows the differences of the amount of NH_4_^+^ and NH_3_ release by pure AlN powder and 20 wt. % of CA/AlN composite. The results clearly show the release of NH_4_^+^ and NH_3_ is controlled by the CA substrate and the generation of aluminum hydroxide on the surface when exposed with water as shown in Equations (2)–(5) play a role to slow down the release. Therefore, the level of toxicity is much lower when compared to pure AlN powder. Secondly, the intended application of these CA/AlN composite is not for ingestion or biomedical devices: it is intended to be used as tablecloth or food wraps which are not in direct contact with the human body environment.

## 5. Conclusions 

The surface characterization of the composite material shows an increment in surface roughness due to presence of AlN, that could also be identified by Raman.

The mechanical strength of the composite was reduced at AlN fractions >10 wt. %. On the other hand, the Young’s modulus showed an increase up to 10 wt. % and a decrease at a higher concentration. In addition, a clear decrement observed in toughness upon increasing concentration of AlN was observed.

The melting temperature (T_m_) and glass transition temperature (T_g_) increased with increasing AlN concentration, showing that the thermal properties of the CA/AlN composites were improved in the presence of AlN.

The CA/AlN composites showed antibacterial effects for both the Gram-positive and Gram-negative bacteria at higher concentration of AlN due to the reaction of AlN with water to produce ammonia (NH_3_) and ammonium ions, which caused lysis by disruption of bacterial cell membrane.

In conclusion, this could be a promising material to replace plastic bags, food packaging or other plastic products thanks to improved antibacterial and thermal property. 

Future research will focus on the degradability and stability of this composite material. 

## Data Availability

The data presented in this study are available on request from the corresponding author.

## Supplementary Materials

The following are available online at https://www.mdpi.com/article/10.3390/antibiotics10111292/s1, Figure S1: Microscopic images of Staphylococcus epidermidis biofilm formation on the different CA/AlN composites after 12 h (a–e), 24 h (f–j), Figure S2: Microscopic images of Escherichia coli biofilm formation on the different CA/AlN composites after 12 h (a–e), 24 h (f–j), Figure S3: Quantification of Ammonium ion and Ammonia released during hydrolysis process of AlN at different time interval, Figure S4: Comparison of Ammonium ion and Ammonia released by AlN powder and 20 wt. % CA/AlN composite.
