# Supplementary material for: Antibacterial Property of Cellulose Acetate Composite Materials Reinforced with Aluminum Nitride
Source: Antibiotics (Basel). 2021 Oct 22;10(11):1292. doi: 10.3390/antibiotics10111292 (PMC8637303; doi:10.3390/antibiotics10111292)
Supplement: Supplementary file 1 [file antibiotics-10-01292-s001.zip › antibiotics-1409783-supplementary.pdf]

antibiotics

MDPI

Article

Antibacterial Property of Cellulose Acetate Composite Materials Reinforced with Aluminium Nitride

Thefye P. M. Sunthar [1,2,] *, Francesco Boschetto [1,3], Hoan Ngoc Doan [5], Taigi Honma [1], Kenji Kinashi [5], Tetsuya Adachi [3], Elia Marin [1,3], Wenliang Zhu [1] and Giuseppe Pezzotti [1,2,4,6]

1 Ceramic Physics Laboratory, Kyoto Institute of Technology, Sakyo-ku, Matsugasaki, Kyoto 606-8585, Japan; thefyeprasad@rocketmail.com (T.P.M.S.); boschetto-cesc@kit.ac.jp (F.B.); m0672026@edu.kit.ac.jp (T.H.); elia-marin@kit.ac.jp (E.M); wlzhu@kit.ac.jp (W.Z.); pezzotti@kit.ac.jp (G.P.)
2 Department of Immunology, Graduate School of Medical Science, Kyoto Prefectural University of Medicine Kamigyo-ku, 465 Kajii-cho, Kawaramachi dori, Kyoto 602-0841, Japan.
3 Department of Dental Medicine, Graduate School of Medical Science, Kyoto Prefectural University of Medicine, Kamigyo-ku, Kyoto 602-8566, Japan; t-adachi@koto.kpu-m.ac.jp (T.A.)
4 The Center for Advanced Medical Engineering and Informatics, Osaka University, Yamadaoka, Suita, Osaka 565-0871, Japan
5 Faculty of Material Science and Engineering, Kyoto Institute of Technology, Sakyo-ku, Matsugasaki, Kyoto 606-8585, Japan; ngochoandoan@gmail.com (H.N.D.); kinashi@kit.ac.jp (K.K.)
6 Department of Orthopedic Surgery, Tokyo Medical University. 6-7-1 Nishi-Shinjuku, Shinjuku-ku, Tokyo 565-0871, Japan.
* Correspondence: thefyeprasad@rocketmail.com

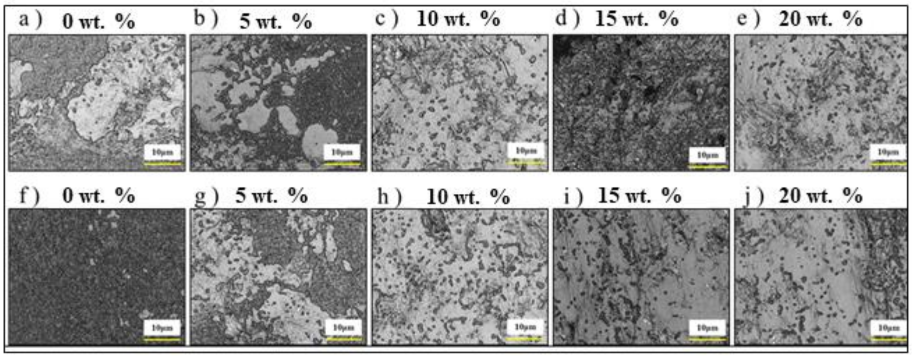

Figure S1. Microscopic images of *Staphylococcus epidermidis* biofilm formation on the different CA/AlN composites after 12 h (a-e), 24 h (f-j)

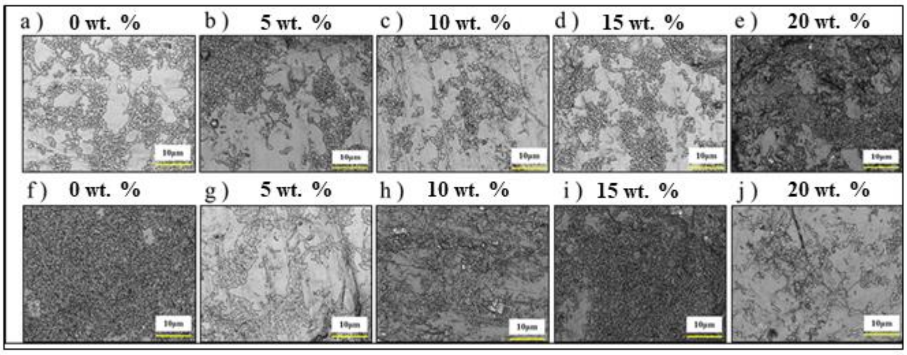

Figure S2. Microscopic images of *Escherichia coli* biofilm formation on the different CA/AlN composites after 12 h (a-e), 24 h (f-j)

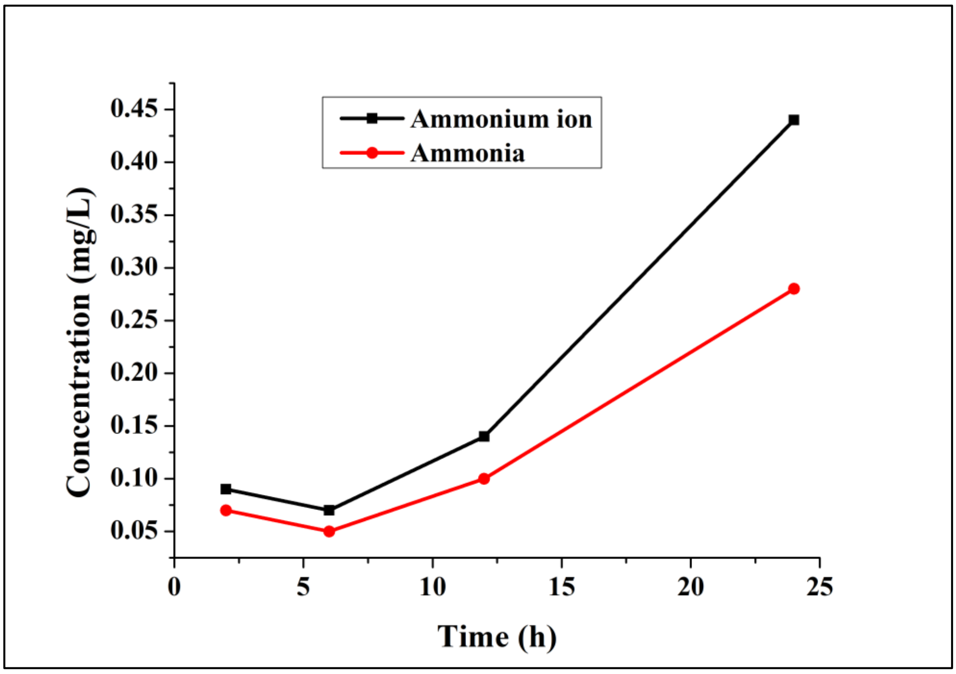

Figure S3: Quantification of Ammonium ion and Ammonia released during hydrolysis process of AlN at different time interval.

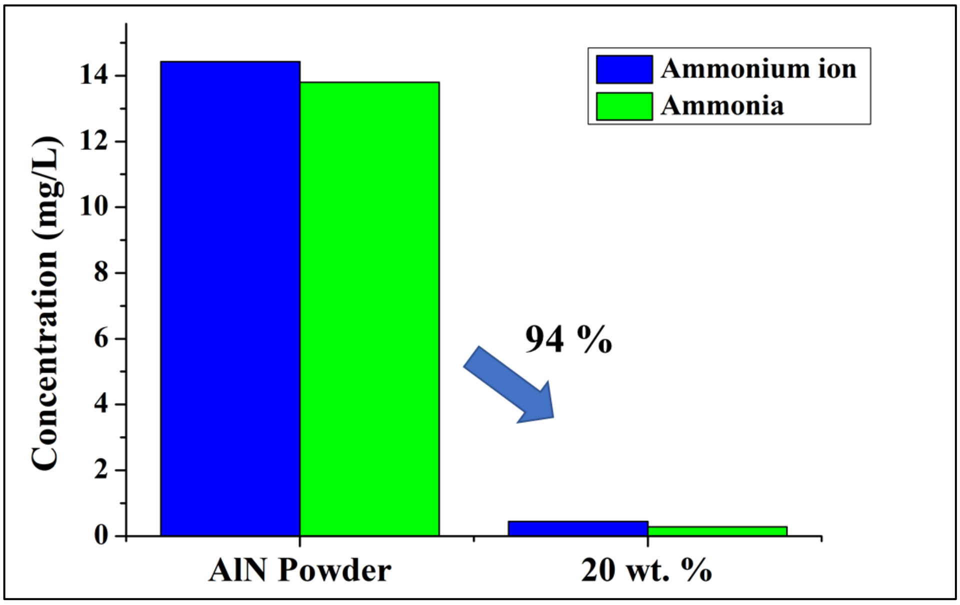

Figure S4: Comparison of Ammonium ion and Ammonia released by AlN powder and 20 wt. % CA/AlN composite.
